# Profiles of alternative splicing events in the diagnosis and prognosis of Gastric Cancer

**DOI:** 10.7150/jca.46239

**Published:** 2021-03-19

**Authors:** Chunyin Wei, Weishun Xie, Xiaoliang Huang, Xianwei Mo, Zujun Liu, Guo Wu, Yongsheng Meng, Franco Jeen, Lianying Ge, Lihua Zhang, Lixian Liao, Jungang Liu, Weizhong Tang

**Affiliations:** 1 Department of Gastrointestinal Surgery, Guangxi Medical University Cancer Hospital, Nanning, Guangxi Zhuang Autonomous Region 530021, P.R. China.; 2 Guangxi Clinical Research Center for Colorectal Cancer, Nanning, Guangxi Zhuang Autonomous Region 530021, P.R. China.; 3 Department of Medical Oncology, Guangxi Medical University Cancer Hospital, Nanning, Guangxi Zhuang Autonomous Region 530021, P.R. China.

**Keywords:** alternative splicing, prognostic signature, gastric cancer, survival, TCGA

## Abstract

**Background:** Gastric cancer (GC) is a heterogeneous disease, and alternative splicing (AS) is a powerful universal transcriptional regulatory mechanism that contributes to the occurrence and development of cancer. However, the systematic analysis of AS events in GC is lacking; therefore, further studies are needed.

**Methods:** Genome-wide analysis of AS events was performed using RNA-Seq data to evaluate the difference between GC and adjacent tissues at the AS level. Prognostic signatures based on differentially expressed alternative splicing (DEAS) events and a correlation network between DEAS and genes were built.

**Results:** We identified 48,141 AS events, of which 2325 showed differential expression patterns. The parental genes before DEAS events play an essential role in regulating GC-related processes such as ribosome (FDR < 0.0001) and thermogenesis (FDR = 0.0002). There were 76 survival-associated DEAS cases. Stratifying patients according to the percent spliced in index value of six types of splicing patterns formed significant Kaplan-Meier curves in the overall survival analysis. A prognostic feature based on DEAS performed well for stratification in patients with GC.

**Conclusion:** The present study will enrich our understanding regarding the distinction of GC and provide a generous amount of biomarkers and potential targets for the treatment of GC.

## Introduction

According to the 2018 global cancer statistics, gastric cancer (GC) is one of the world's deadliest cancers with over 1,000,000 new cases and 783,000 estimated deaths in 2018, ranking third-highest in mortality rate 1. In China, GC is the third most common malignant tumor and second-ranking in mortality rate, and the incidence of GC increases annually 2. Although progress has been made in diagnoses and multidisciplinary treatments, the overall five-year survival rate of patients diagnosed with GC ranges from 25% to 27%, and the survival rate of patients with advanced disease is even lower 3. Over the past few decades, research has shown that the occurrence, recurrence, and metastasis of GC is the result of complex interactions between the host and environmental factors, including phenotypic complexity, multiple factors, and multistep processes affected by genetic heterogeneity and ethnic diversity 4. Therefore, understanding the relationship between the biological mechanism of GC regulation and the corresponding clinicopathological features is an important step for targeted therapy and improvement of the quality of life of GC patients.

With the rapid development of high-throughput technology, a new era has opened for cancer genomics studies. Alternative splicing (AS) is a vital process involved in RNA transcription. In AS, a single pre-messenger RNA is spliced into different permutations to generate the structure and function of mRNA 5. Gene is one of the basic codes of gene expression. Genes are first transcribed into pre-mRNA that contain exons and introns. When pre-mRNA is spliced into mRNA, AS removes introns and connects exons. Alternative mRNA transcripts are thus produced by the retention or exclusion of various exons and introns 6, 7. Studies show that the number of protein-coding genes is less than 25,000, which is much lower than the number predicted by about 100,000 protein-coding genes in the complexity of the human proteome 8, 9. AS accounts for the difference between the number of protein-coding genes and proteins and significantly increases the proteome diversity and cell complexity 10. More than 90% of human genes undergo AS 11, and AS affects protein function by adding or removing domains, modifying protein-protein interactions, and changing protein stability 12. In recent years, extensive genomic and functional studies firmly established the role of AS in cancer 13, 14. Particularly, widespread aberrant AS is a molecular marker of tumorigenesis 15. AS exists in a variety of carcinogenic processes such as cell proliferation, apoptosis, hypoxia, metabolism, angiogenesis, and immune escape 16, 17. In addition, AS abnormalities are potential biomarkers for tumor occurrence and prognosis as well as therapeutic targets for malignant tumors 17.

The role of AS in human diseases, especially cancer, is widely recognized 6, 13, 18-20. However, due to technical limitations, there is little research on the role or function of AS events in GC. Nevertheless, recent approaches using high-throughput techniques have been successful in GC. For example, one study showed that PTBP3 as a GC metastasis gene regulated CAV1 through AS 21. Another study demonstrated that H3K36me3, in the region associated with low-level histone acetylation and histone methylation, was related to exon exclusion in the region 10-11 of the hMLH1 exon, which suggested that histone-based AS regulation may be involved in the identification of AS sites in GC 20. Both those studies lacked relevant clinical information 20, 21, which hindered the systematic annotation of the clinical significance of GC-specific AS events.

The Cancer Genome Atlas (TCGA) project provides a rich source of data for the study of the AS mode in cancer, including data on the expression levels of exons, splicing, and transcriptional isomers. To date, highly efficient and reliable bioinformatics processing technology is used to address low-voltage and complex AS events 22. Therefore, it is possible to study the clinical effects of tumor-related AS events in the population by obtaining data from TCGA and using bioinformatics technology. For example, 421 differentially expressed alternative splicing (DEAS) events were found in colorectal cancer The parent genes before DEAS have protein kinase activity, the signaling pathways of p53 and PI3K-Akt 19. However, a comprehensive analysis of survival with the individual exon resolution associated with AS is lacking in tumors, especially in GC. Therefore, we systematically analyzed genome-wide AS in a GC cohort from TCGA, identified GC-related AS events, and investigated their relationship to clinical outcomes. A series of prognostic AS were recognized, and high-efficiency prognostic characteristics were built according to distinct AS.

## Materials and methods

### Clinical specimens

Thirty GC patients who underwent gastrectomy at Guangxi Medical University Cancer Hospital from May to November 2020 were enrolled in this study. All enrolled patients had primary GC. None of the patients underwent chemotherapy or radiotherapy prior to tissue collection, and the specimens collected were GC and adjacent tissues.

This study was approved by the Ethics and Human Discipline Committee of Guangxi Medical University Cancer Hospital. Written informed consent was obtained from all patients. All experiments and methods were in accordance with the relevant guidelines and regulations.

### Data acquisition and processing

We downloaded AS data from TCGA SpliceSeq, which is a network-based resource for exploring the AS patterns of 33 different tumor types 23. The level 3 RNA-Seq and clinical data for GC were downloaded from the Genomic Data Commons Data Portal. The percent spliced in index (PSI) quantifies AS events as a value ranging from 0 to 1 22. To generate a reliable set of AS events, a series of stringent filters is necessary (AS events occurring in ≥ 75% of samples and the average PSI value ≥ 0.05). RNA-Seq, AS, and clinical data have matching TCGA barcodes. The inclusion criteria for patients in this study were as follows. (1) The clinical parameters available for the patient included sex, age, tumor location, lymph node and distal metastasis, pathological stage, and survival information. (2) AS and RNA-Seq data were available for the patient. The exclusion criteria for patients were as follows. (1) The clinical parameters available for the patient were incomplete. (2) The patient was diagnosed with malignant tumors other than GC. We downloaded AS and clinical data from the TCGA database for 452 GC patients including 34 from adjacent and 418 from GC tissues. After screening for the inclusion and exclusion criteria, 34 adjacent and 367 GC tissues were included in the study.

### Identification of differentially expressed alternative splicing (DEAS) events and enrichment analysis

To identify DEAS in GC, the Benjamin-Hochberg method was used to adjust the P-value in the t-test. Considering the small PSI value, we set the significance level for identifying DEAS at P < 0.05. The ClusterProfler package was used to carry out the GO and Kyoto encyclopedia of genes and genomes (KEGG) enrichment analysis of DEAS parental genes 24. Items with a P-value < 0.05 were selected for further analysis.

### Survival analysis

A total of 367 GC patients with complete follow-up information and AS data were enrolled in the survival analysis. Clinical data included age, sex, local invasion, lymph node and distant metastasis, TNM stage, and survival status (Table S1). For each DEAS event, a univariate Cox regression was performed according to the PSI value. Then, the DEAS input multivariable Cox regression with p < 0.05 in univariate Cox regression. Multivariate Cox regression was performed according to different AS patterns to establish the prediction models. The independent DEAS in the final multivariate Cox regression was screened from the multivariate Cox regression of different AS patterns. The final prognostic model was constructed based on the independent DEAS from the final multivariate Cox regression. We used a Kaplan-Meier curve to evaluate whether the prognostic model could distinguish between subgroups of GC patients with good or poor prognosis. The identification of each prognostic model over five years (1825 days) was further evaluated by receiver operating characteristic (ROC) curves using the survival ROC package.

### Gene interaction network analysis

We downloaded the parent genes related DEAS from the Retrieval of Interacting Genes/Proteins (STRING) 9.1 database 25. The interaction score for the protein interaction network generated in STRING was set to 0.9, and all other settings were set to default parameters. The gene interaction network was visualized by Cytoscape v3.4.0 26.

### RT-qPCR validation of AS events in gastric cancer (GC)

Differential expression of AS events was validated using RT-qPCR. Total RNA was extracted using Trizol reagent (Invitrogen, USA). The cDNA was reverse transcribed using 2-6 μg of total RNA with the M-MLV reverse transcriptase (Promega, USA). RT-qPCR was performed on a qTOWER3 G real-time PCR system (Analytik Jena, Germany). The total reaction volume was 20 μL and consisted of 0.1 μM of each primer 10 μL of GoTaq® qPCR Master Mix (Promega, USA), and 20-100 ng of cDNA. The PCR reaction condition was as follows: 95 °C for 10 min, then 40 cycles of 95 °C for 15 s, and 60 °C for 1 min.

We chose a method similar to the PSI value calculation to quantify the expression of a specific AS event in GC and adjacent tissues. Two pairs of primers (Table S2) were used to amplify the AS isoforms and common transcripts. Data normalization with the reference GAPDH gene expression and the 2 -∆∆CT method was used to calculate the relative expression of each gene.

## Results

### DEAS events in GC

**Figure 1:**
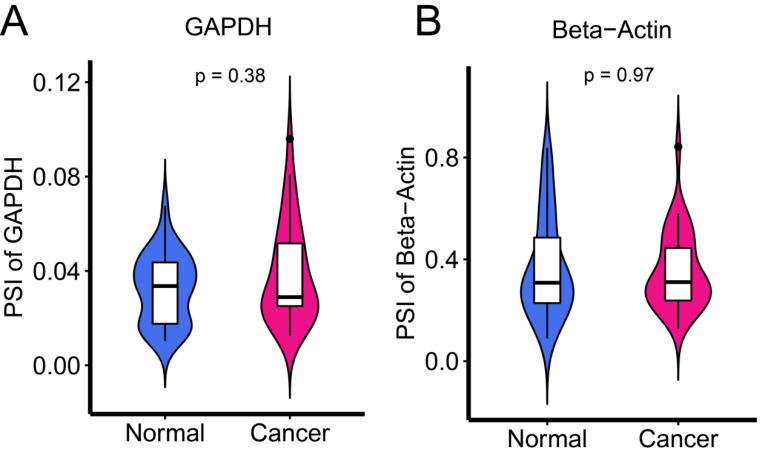
Internal reference genes GAPDH and Beta-Actin to examine the expression of AS levels in cancer and adjacent tissues.

To examine the expression levels of AS in cancer and adjacent tissues, the common internal reference genes, GAPDH and β-actin, were applied. There was no significant difference between the two groups (Figure 1) (all P > 0.05).

**Figure 2:**
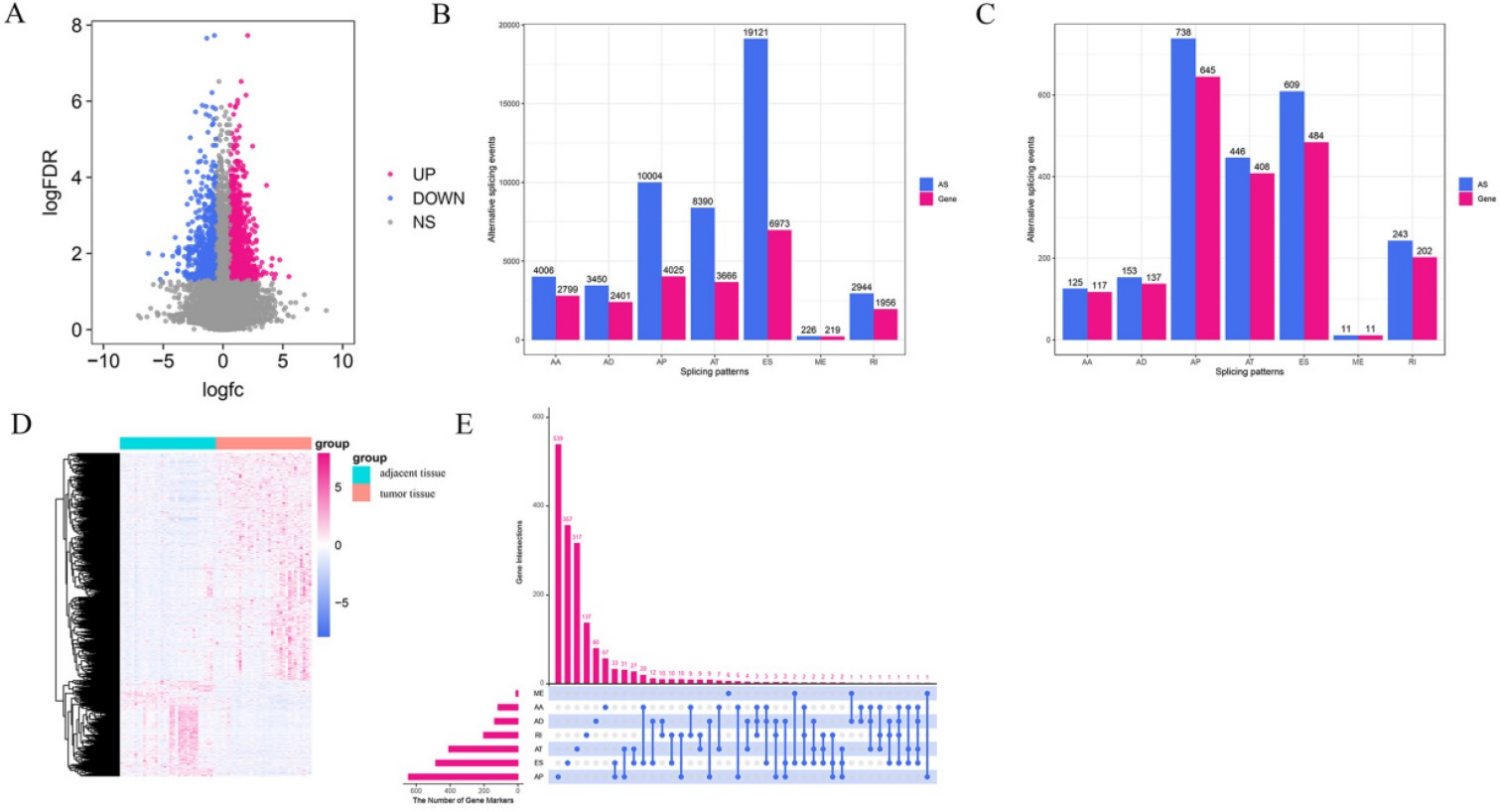
** Overview of AS events profiling in gastric cancer**. (A) Volcano plot shows that all the differentially spliced AS (DEAS) events have different levels of expression in the tumor tissues and the adjacent tissues. The log FC indicates the logarithmic conversion multiple of the PSI value of DEAS. (B) The number of AS events and the number of genes associated with each AS type in 367 with gastric cancer patients. (C) Upregulated of DEAS events in gastric cancer. (D) Heatmap of DEAS events in tumor tissues and the adjacent tissues. (E) UpSet plot showing the interactions between the seven patterns of DEAS increased in gastric cancer. Seven types of AS events, including alternate acceptor site (AA), alternate donor site (AD), alternate promoter (AP), retained intron (RI), exon skip (ES), and mutually exclusive exons (ME).

Integrated AS event profiles were explored in depth for 367 GC patients. In total, 48,141 AS events from 22,039 genes remained to be further analyzed. There were seven types of splicing patterns including 19,121 exon skip (ES) events in 6,973 genes, 226 mutually exclusive (ME) exon events in 219 genes, 2,944 retained intron (RI) events in 1,956 genes, 10,004 alternate promoter (AP) events in 4,025 genes, 8,390 alternate terminator (AT) events in 3,666 genes, 3,450 alternate donor site (AD) events in 2,401 genes, and 4,006 alternate acceptor site (AA) events in 2,799 genes (Figure 2B). The ratio of AS events to genes was approximately 2, indicating an average of two events per gene. Furthermore, the results demonstrated that half the AS events were ES. All the above AS events were quantitated according to the PSI value, which is typically used to quantify AS events. Some splice isomers had extremely low expression levels (PSI < 0.05). Therefore, to obtain a set of reliable GC AS events, >75% of the samples were classified as AS events and the average PSI was >0.05.

To discern any differences in the expression levels of AS, we compared the expression of AS in 34 pairs of matched GC and adjacent tissues. A total of 2,325 DEAS events in 2,004 genes were identified (Figure 2C), which accounted for 4.83% of the AS events. On average, one gene may have one or more AS events, indicating a diverse involvement of DEAS events in GC. Considering that, we used the UpSet plot to visualize the intersection sets of each AS type (Figure 2E). There were only a few genes that had more than one type of AS event and were differentially spliced in GC. The volcano plot visualized the DEAS events identified in GC (Figure 2A). All DEAS events were expressed at different levels in GC and adjacent tissues, which were statistically significant (adjusted P-value < 0.05, |logFC| ≥ 1) (Figure 2A). On this basis, an unsupervised hierarchical clustering method was used to divide the GC and adjacent tissues into two groups. The screened DEAS was credible (Figure 2D). The results suggest that the relationship between GC-related AS events and GC biology needs to be further researched in the future.

### Enrichment and interaction analysis of DEAS events

**Figure 3:**
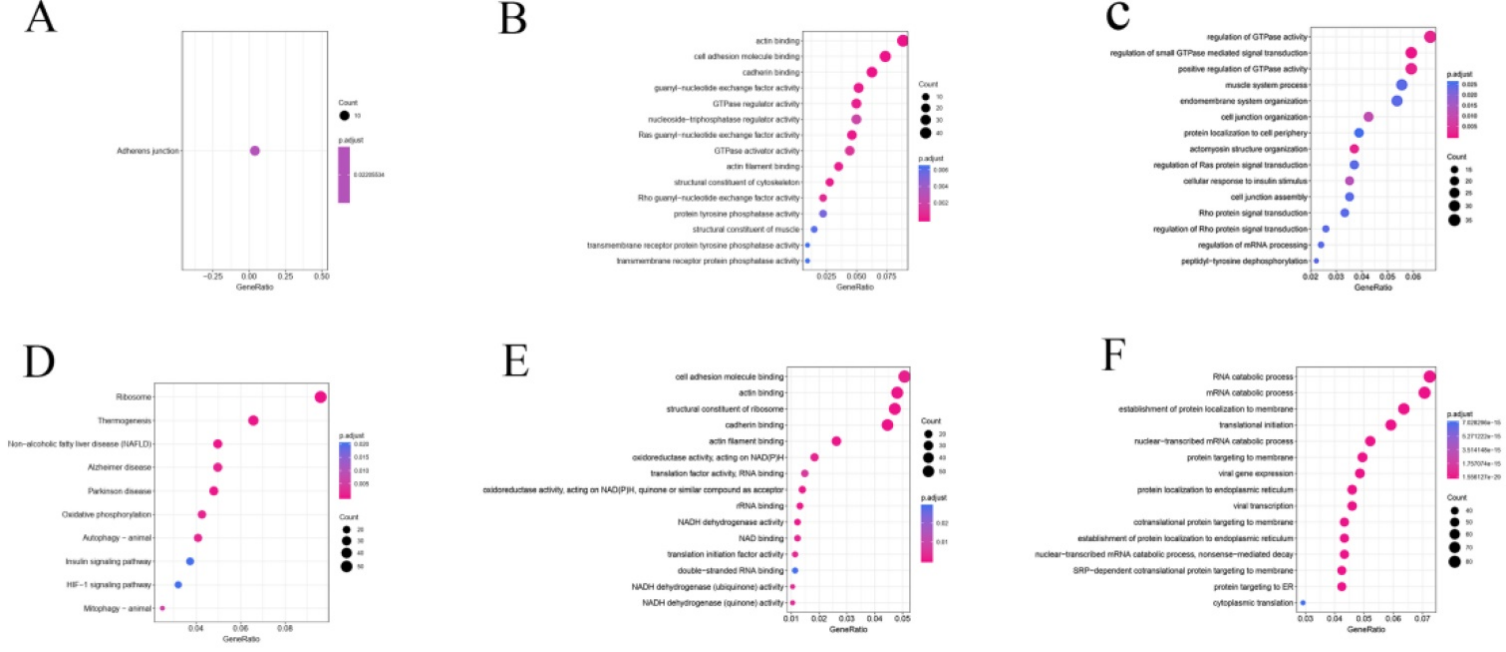
**Functional KEGG analysis and GO analysis of differentially spliced genes.** (A and D) KEGG pathway. (B and E) GO molecular function. (C and F) GO biological process.

AS may directly regulate protein function through a variety of mechanisms. Hence, we carried out an enrichment analysis based on differential splicing genes (DSGs) to explore the potential function and pathways of DEAS. In the KEGG pathway analysis, DEAS increased when the KEGG pathway was hypoexpressed in GC, which was significantly enriched at the adherens junction (P = 0.02) (Figure 3A). DEAS increased when the KEGG pathway was hyperexpressed in GC, which was significantly enriched in ribosome (P = 4.5×10^-22^) and thermogenesis (P = 1.8×10^-4^) (Figure 3D). In GO molecular function (MF) enrichment analysis, actin (P = 1.9×10^-12^) and cell adhesion molecule binding (P = 8.0×10^-6^) were enriched in the hypoexpression of MF in GC (Figure 3B), whereas cell adhesion molecule (P = 1.3×10^-3^) and actin binding (P = 2.8×10^-4^) were enriched in the hyperexpression of MF in GC (Figure 3E). In GO biological process (BP) enrichment analysis, regulation of GTPase activity (P = 1.8×10^-3^) and small GTPase-mediated signal transduction (P = 1.7×10^-8^) were enriched in the hypoexpression of BP in GC (Figure 3C), whereas RNA (P = 7.4×10^-20^) and mRNA catabolic processes (P = 4.5×10^-21^) were enriched in the hyperexpression of BP in GC (Figure 3F).

### Survival-associated DEAS events in GC

**Figure 4:**
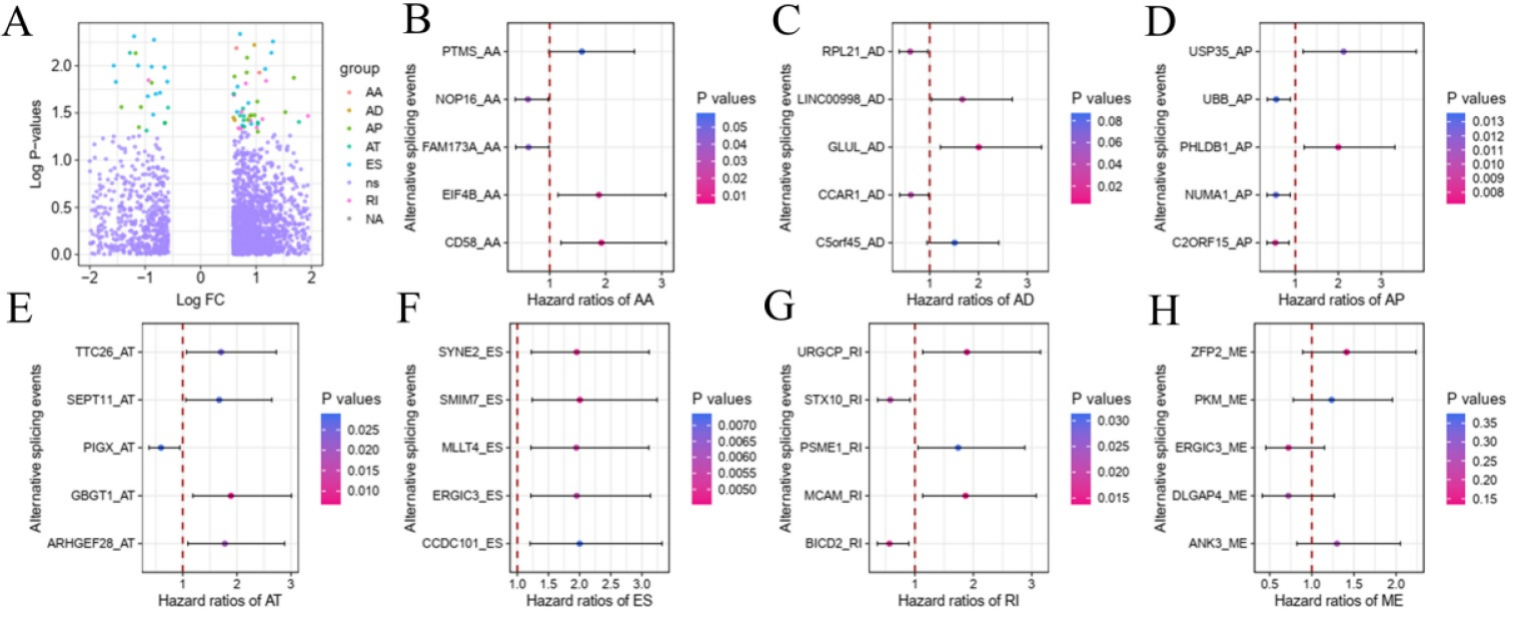
** Forest plots of hazard ratios for survival-related DEAS of subgroup analyses in gastric cancer.** (A) The volcano plot depicts the P-values of the univariate Cox regression of the 2325 DEAS in 367 patients. (B-H) forest plots of HRs for top5 smallest P-values AS events for seven splicing patterns AA, AD, AP, AT, ES, RI and ME, respectively. The P-values are represented according to the color scale of the side. Horizontal bars represent 95% CIs.

**Figure 5:**
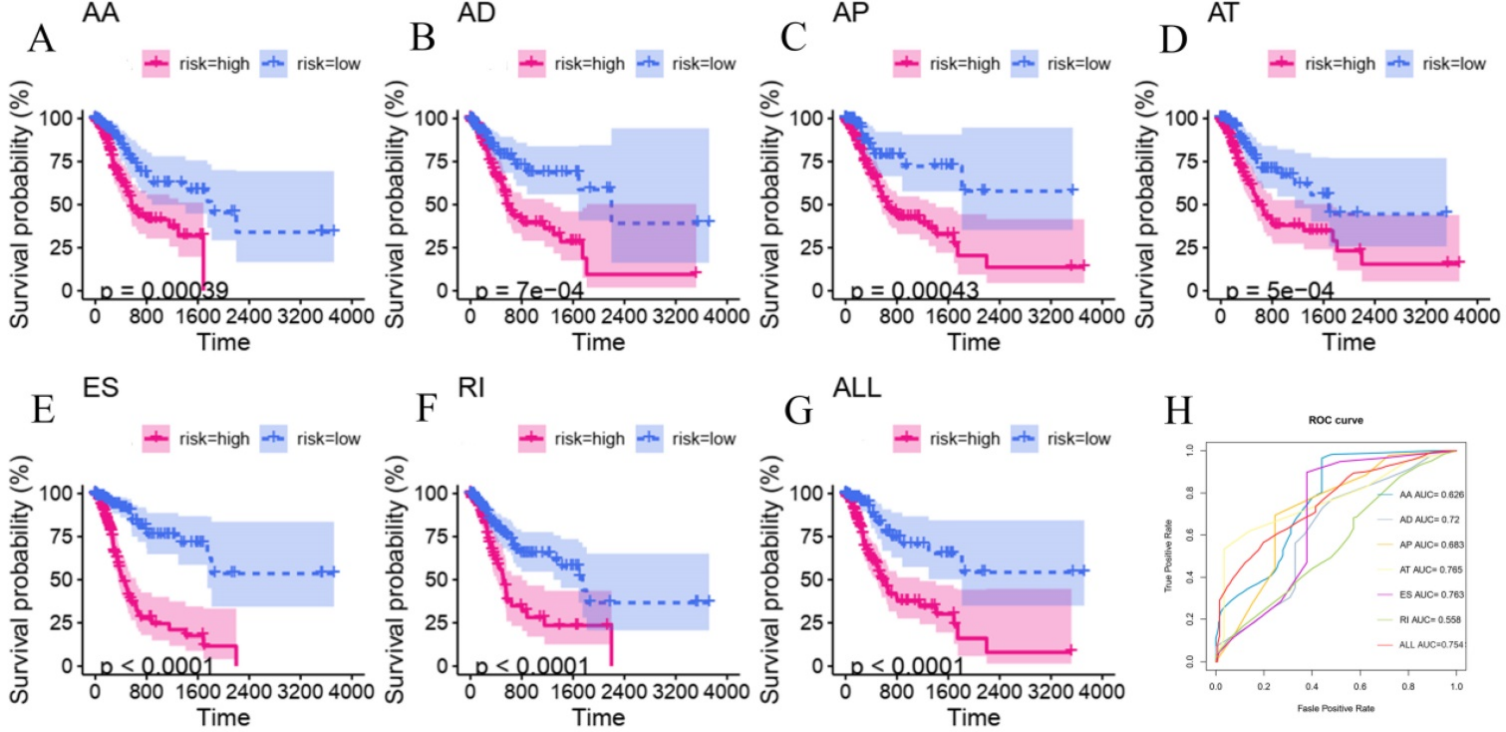
** Kaplan-Meier and ROC curves of prognostic predictors for gastric cancer.** (A-F) Kaplan-Meier curves of prognostic predictor models constructed according to six AS patterns including AA, AD, AP, AT, ES and RI. The blue line represents the trend of the low-risk group, while the red line represents the trend of the high-risk group. (G) ROC curves of predictive models with different AS patterns.

**Figure 6:**
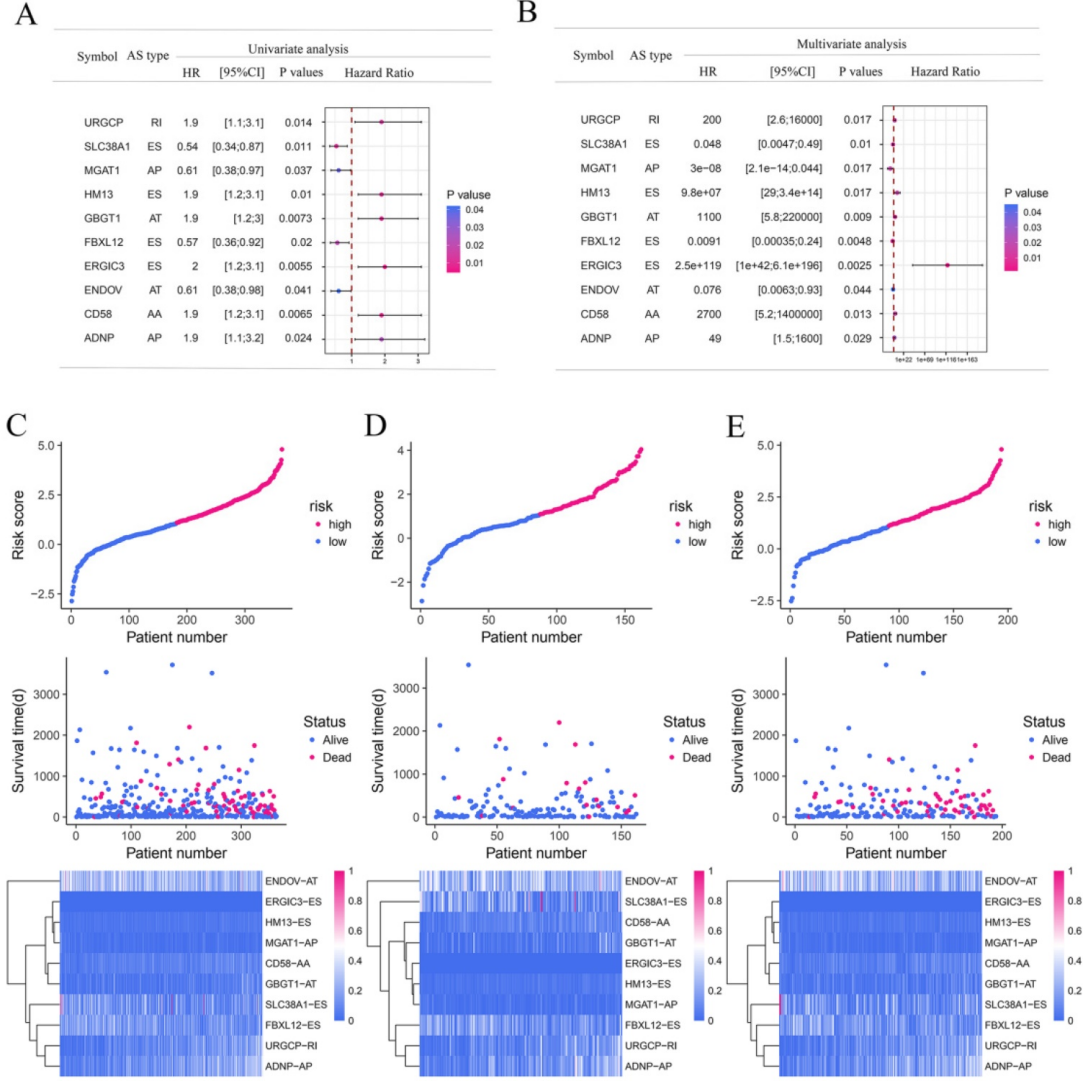
** The prognostic predictors' value of the DEAS signature in gastric cancer.** (A) Univariate analysis of the 10 overall survival predictive factors. The P-values are represented according to the color scale of the side. Horizontal bars represent 95% CIs. (B) Multivariate analysis of the 10 overall survival predictive factors. The P-values are represented according to the color scale of the side. Horizontal bars represent 95% CIs. (C-E) Risk score analysis of gastric cancer patients. The top groups indicated the risk scores of the patients. The middle groups indicated that the survival status and duration of patients were distributed according to the risk score. The bottom groups indicated that the PSI values of the 10 predictive factors were distributed according to the risk score in the heatmap. (C) All gastric cancer patients. (D) Stage I-II for gastric cancer patients. (E) Stage III-IV for gastric cancer patients.

To explore the underlying relationship between DEAS and overall survival (OS) in GC, we performed a univariate Cox regression of 2,325 DEAS in 367 patients. As shown in Figure 4A, 76 survival-associated DEAS events were identified (P < 0.05), which accounted for 0.16% of the AS events. AA, AD, AP, AT, ES, and RI contained survival-associated DEAS. Survival-associated DEAS was most common in ES (25 cases), followed by AP (18 cases). Survival-associated DEAS was least common in AA and AD (4 cases). For each splicing pattern, the hazard ratios (HRs) of five AS events with the smallest P-values were selected (Figure 4B-H). Then, to identify independent prognostic DEAS in GC, we used a single factor Cox regression for preliminary screening and the filter (P < 0.05 in the univariate Cox regression) for selecting variables for the multivariate Cox regression. Six types of splicing patterns were analyzed using multivariate Cox regression. Next, six different types of independent prognostic DEAS were combined to construct the final prognostic predictor. The multivariable Cox regression results for each AS pattern are shown in Figure 5A-5F, and all AS patterns are shown in Figure 5G. The GC patient grouping was based on the median risk score predicted by the prognostic model, which was divided into high-and low-risk groups. Six prognostic models were capable of predicting the prognosis of patients with GC. The prognostic models based on ES, RI, and ALL AS patterns were the most significant factors affecting prognosis (P < 0.0001). To further evaluate the discriminability of these prediction models, ROC curves were drawn and the area under the curve (AUC) was calculated (Figure 5H). In the prognostic model, AT demonstrated the best discrimination with an AUC of 0.765, followed by ES with an AUC of 0.763 and ALL with an AUC of 0.754. To obtain the final prognostic model, a multivariate Cox regression was used to evaluate the independent prognostic DEAS in each splicing pattern. Ten DEAS events were identified as independent prognostic factors in the multivariate Cox regression, and these ten DEAS events were used to construct the final prognostic model. The HRs and P-values of DEAS events with independent prognosis in the 10 cases are shown in Figure 6A & B. Risk score analyses indicated that the final prognostic model showed a significant ability to distinguish between good and poor prognosis in GC patients (Figure 6C). In the final prognostic model, subgroup analysis indicated that the model could effectively distinguish between good and poor outcomes in patients with stage I-IV GC (Figure 6D-E). The expression of 10 independent prognostic DEAS events in GC is shown in Figure S1. Details of the 10 AS events in the prognostic model are shown in Table 1.

### Validation of survival-associated DEAS events in GC

**Figure 7:**
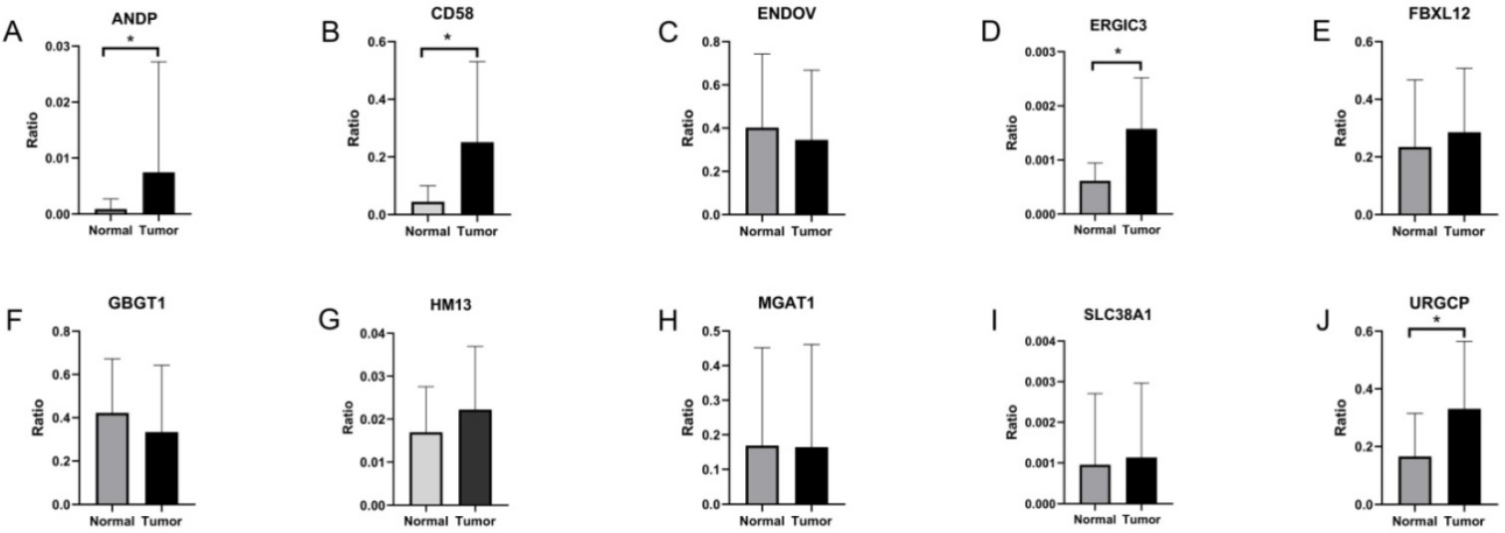
Validation of the 10 survival-associated DEAS events in gastric cancer. *P < 0.05.

To validate survival-associated DEAS expression in GC, RT-qPCR was used. The expression of ten independent prognostic AS events in GC and adjacent tissue specimens was assessed. The expression of ANDP, CD58, ERGIC1, and URGCP was significantly higher in GC than in adjacent tissues (P < 0.05) (Figure 7A, B, D & J). On the other However, the expression of ENDOV, FBXL12, GBGT1, HM13, MGAT1, and SLC38A1 did not differ significantly between GC and adjacent tissues (Figure 7C, E, F, G, H & I).

### Gene interaction network of DEAS

Because of the structural variation (AS) in gene transcripts, it is possible that protein translation will be affected, thereby modifying the characteristics of the proteins 27, 28. Therefore, it is very important to understand the interaction between DSGs from the perspective of the protein network. The DSG-based protein-protein interaction (PPI) network analysis shows DSG interactions in GC (Figure S2). The network consists of 29 nodes and 30 edges. RPL21, UBB, and SKP1 were identified as hub genes in the network, which suggested that the AS of ribosomal proteins and ubiquitin-proteasomes were implicated in the tumorigenesis and development of GC.

## Discussion

GC is a disease characterized by the complex interactions between host and environmental factors, including phenotypic complexity, multiple factors, and multistep processes affected by genetic heterogeneity and ethnic diversity 4. Although molecular differences in GC at genomic and epigenetic levels have been partially revealed, there are still areas that remain unknown. AS is the main mechanism for controlling gene expression and determines the complexity of the cell and the diversity of the proteome. Aberrant AS is widely accepted as a contributor to the occurrence, development, and metastasis of cancer 29, 30. A comprehensive analysis of AS expression in GC is of great significance. The present study identified a significant correlation between AS and the clinical outcomes of GC. A total of 48,141 AS events were detected in 22,039 genes. There were 65,152 AS events in breast cancer, 70,342 AS events in liver cancer, and 70,637 AS events in lung cancer 31. Compared with the AS events reported in GC in the present study, the number of AS events in the other cancer types were much higher than that in GC, possibly because of the low mutation rate in GC 31. In addition, 2,325 DEAS events and 2,004 genes were associated with GC, and 76 of those DEAS events were associated with OS. A prognostic feature including 10 survival-related DEAS events was built. In general, there are seven types of AS patterns including AA, AD, AP, AT, ES, ME, and RI. The most common AS pattern in both vertebrates and invertebrates is ES, which accounts for approximately 30% of all AS patterns 32. However, in this study, AP (31.7%) was the most common AS pattern, followed by ES (26.2%), which may be related to the heterogeneity and epigenetics of GC but this relationship needs further study. Moreover, an interaction network between the gene and DEAS was constructed, which provided the basis for a comprehensive understanding of the function of AS in GC.

In the enrichment analysis, all three enrichment analyses were associated with cancer, which indicated that DEAS was involved in the tumorigenesis and progression of GC.

In the KEGG pathway analysis, adherens junction was the most significant pathway in the hypoexpression of KEGG pathway. The adherens junction is involved in many biological processes such as epithelial monolayer bending, collective cell migration, cell extrusion, and wound healing 33-35. The enrichment analysis of cholangiocarcinoma revealed an increase in adherent function in the disease 36. However, our study showed that the adherens junction was increased when the KEGG pathway was hypoexpressed; therefore, regardless of whether the expression of the adherens junction increases or decreases, it can cause cancer. Moreover, ribosome and thermogenesis were the most significant pathways when the KEGG pathway was hyperexpressed. An increase in ribosome when the KEGG pathway was hyperexpressed was reported in colorectal cancer 37, but there was a lack of research on the relationship between thermogenesis and all cancers. In GO molecular MF enrichment analysis, actin and cell adhesion molecule binding were the most significant pathways in the hyperexpression and hypoexpression of MF; both are closely associated with oncogene expression, tumor metastasis, and recurrence 38-40. In GO BP enrichment analysis, regulation of GTPase activity and small GTPase-mediated signal transduction were the most significant pathways in the hypoexpression of BP; both have been linked to cancer progression 41, 42. One study showed that small GTPase-mediated signal transduction in glioblastoma cell apoptosis can be induced by excessive expression of RND3 42. In addition, RNA and mRNA catabolic processes were the most significant pathways in the hyperexpression of BP. The synthesis of RNA and mRNA is a key step in transcription and translation 5. If mutations or errors occur, they can lead to protein synthesis errors and even diseases, including cancer 43. RNA and mRNA catabolic processes are key in detecting and eliminating improperly processed cell RNA 44, 45. Our results indicated that regardless of whether the expression of DEAS was increased or decreased, the immune-related pathways were implicated in the tumorigenesis of GC.

To evaluate the potential value of specific DEAS events as a prognostic indicator of GC, we established a prognostic model for a single AS pattern. The results showed that the ES pattern was the most effective in assessing the survival outcome of patients with GC. In addition, the ideal prognostic model was a combination of all AS patterns. The 10 prognostic factors in the final prognostic model included URGCP-RI, SLC38A1-ES, MGAT1-AP, HM13-ES, GBGT1-AT, FBXL12-ES, ERGIC3-ES, ENDOV-AT, CD58-AA, and ADNP-AP. URGCP, known as URG 4, is upregulated in many common cancers, including hepatocellular carcinoma, osteosarcoma, epithelial ovarian cancer, and GC 46-49. URGCP is a tumor promoter that promotes the proliferation of GC cells 48, 50. URGCP is related to cell cycle, cell adhesion, apoptosis, transcription, and gene expression 48, 50 and located on chromosome 7 (7p13). Previous data suggested that URGCP was upregulated in both human GC tissues and cell lines 48. Upregulation of URGCP could promote cell proliferation, whereas downregulation of URGCP could inhibit proliferation and tumor formation of GC cells 48. In this study, RI occurred in 4.4:4.5 exons of URGCP and was associated with OS in GC. AS in URGCP may change the structure of proteins, therefore the role of different URGCP isoforms in GC requires further study. SLC38A1 is an amino acid transporter A that is involved in amino acid uptake via small side chains, such as alanine, serine, proline, and glutamine 51, 52. The activity of SLC38A1 is affected by cell volume, pH, glucagon, insulin, and insulin-like growth factor-1 51. The expression of SLC38A1 in GC is closely related to age, TNM stage, PCNA expression, differentiation status, and lymph node metastasis, which can be used as an indicator of disease invasiveness 53. Overexpression of SLC38A1 correlates with poor prognosis 53. However, the expression and function of different subtypes of SATB2 in GC require further study.

Finally, our program succeeded in identifying several AS events in genes and established efficient prognostic models for GC. However, several potential limitations to our study should be mentioned. First, we failed to identify a few typical DEAS events in the development of GC, such as CD44, survivin, and MYH 54. This may be due to the strict inclusion criteria we used. Second, the results of the PPI network analysis need to be validated by molecular biological experiments. Lastly, the clinical specimens used to validate the DEAS events were small; therefore, large clinical specimens are needed to validate the results of this study.

In summary, this study successfully analyzed the common cancer-specific and survivor-related events in GC. A series of prognosis-associated DEAS events were identified. An efficient prognostic model and an interaction network between DEAS and genes were constructed. The present study will enrich our understanding of the distinction of GC and provide a generous amount of biomarkers and potential targets for the treatment of GC.

## Supplementary Material

Supplementary figures and tables.

## Figures and Tables

**Table 1:** The detailed information of the 10 AS events in the prognostic model

Symbol	As id	Splice type	Exons	From exon	To exon
URGCP	79358	RI	4.4:4.5	4.3	4.6
CD58	4363	AA	5.1	4.1	5.2
SLC38A1	21327	ES	1.2:3	1.1	4
FBXL12	47420	ES	4.2	2.4	5
HM13	58890	ES	12.1:12.2	11	13
ERGIC3	59177	ES	9	8	12
ADNP	59790	AP	4.1		
MGAT1	75019	AP	2		
ENDOV	44054	AT	13		
GBGT1	88019	AT	4		

## Abbreviations

AA: alternate acceptor site
AD: alternate donor site
AP: alternate promoter
AS: alternative splicing
AT: alternate terminator
AUC: area under the curve
BP: biological process
DEAS: differentially expressed alternative splicing
DSGs: differentially spliced genes
ES: exon skip
GC: gastric cancer
HRs: hazards ratios
KEGG: Kyoto encyclopedia of genes and genomes
ME: mutually exclusive exons
MF: molecular function
OS: overall survival
PPI: protein interaction
PSI: percent spliced in index
RI: retained intron
ROC: receiver operating characteristic
TCGA: Cancer Genome Atlas
