# Supplementary material for: Profiles of alternative splicing events in the diagnosis and prognosis of Gastric Cancer
Source: J Cancer. 2021 Mar 19;12(10):2982–92. doi: 10.7150/jca.46239 (PMC8040899; doi:10.7150/jca.46239)
Supplement: Supplementary file 1 — Supplementary figures and tables. [file jcav12p2982s1.pdf]

Table S1: Clinical features of gastric cancer patients in TCGA.

Characteristics		No. of patients (%) (n = 443)
Age (years)		
	Median(IQR)	66(30-90)
	< 60	133
	≥ 60	305
Sex		
	Female	158
	Male	285
Local invasion		
	T1–T2	116
	T3–T4	317
	Tx	10
Lymph node metastasis		
	N0	132
	N1	119
	N2	85
	N3	88
	Nx	17
Distant metastasis		
	M0	391
	M1	30
	Mx	22
TNM stage		
	I	59
	II	130
	III	183
	IV	44
Survival status		
	No	356
	Yes	87

[a]Age data were not available for 5 individuals; [b] lymph node metastasis was not available in 2 case; [c]pathologic TNM stage was not available for 27 individuals.

Table S2: The primers used in the study.

Gene symbol		Primers
URGCP	COMMON PRIMER	AAGTGGAATTACTGGGCAAAGG
URGCP	AS PRIMER	ACCTATCAAACTGGCCGCAT
URGCP	TOTAL PRIMER	CCACAGCTGTTCGTCTCTCT
CD58	COMMON PRIMER	TCTCCCACACTAACTTGTGCA
CD58	AS PRIMER	AGAAATTGTCTTCTCAATTAAAGA
CD58	TOTAL PRIMER	TGGTTCTGTCTGGTTTTCTGTCA
SLC38A1	COMMON PRIMER	CCTCGGGCACTGTCATGTTT
SLC38A1	AS PRIMER	GTCCTTTGTCTTGCTCTGCG
SLC38A1	TOTAL PRIMER	AGTGTCTACTGTGTGCCAGG
FBXL12	COMMON PRIMER	CTGTCTCGGTCACACGGTAG
FBXL12	AS PRIMER	TGCCAAGTAGAGAAATCCAGCA
FBXL12	TOTAL PRIMER	GAAGAGGCTGGTGGACGAC
HM13	COMMON PRIMER	TCGGTTTTCCTGTCCTGGTG
HM13	AS PRIMER	TACATGGCGCTGGGATTCTG
HM13	TOTAL PRIMER	ATCTGTCTGGTCTGGCCCT
ERGIC3	COMMON PRIMER	TCGCTGTGAGAGCTGCTATG
ERGIC3	AS PRIMER	CCAGGCTCCGTGCTATCATC
ERGIC3	TOTAL PRIMER	GGAAGTTTCCGGCCACCTTA
ADNP	COMMON PRIMER	CGCTGCAGCAGAAAGGTTTT
ADNP	AS PRIMER	TGCGCGACAGAGTAAGACTC
ADNP	TOTAL PRIMER	GGCAGTTTAAGAAAAGCCCGG
MGAT1	COMMON PRIMER	ACCTCCCTTCATTCAGCTGC
MGAT1	AS PRIMER	GGGTAGAAAAATGGGCGGGA
MGAT1	TOTAL PRIMER	ATGGGCCAGTTCTCACAGTG
ENDOV	COMMON PRIMER	CTGACATCTGCTCCCGAGAG
ENDOV	AS PRIMER	TCCCTTAAGATCAGGAACAAGACA
ENDOV	TOTAL PRIMER	GGAGTCTCCTTTGGGGCATG
GBGT1	COMMON PRIMER	CCTCAGAGACCAGGCTCAGA
GBGT1	AS PRIMER	GTGTGCCTGTAATCCCAGCT
GBGT1	TOTAL PRIMER	GACATAGGAGACTGGCAGCC
GAPDH	FORWORD PRIMER	GCACCGTCAAGGCTGAGAAC
GAPDH	REVERSE PRIMER	GGATCTCGCTCCTGGAAGATG

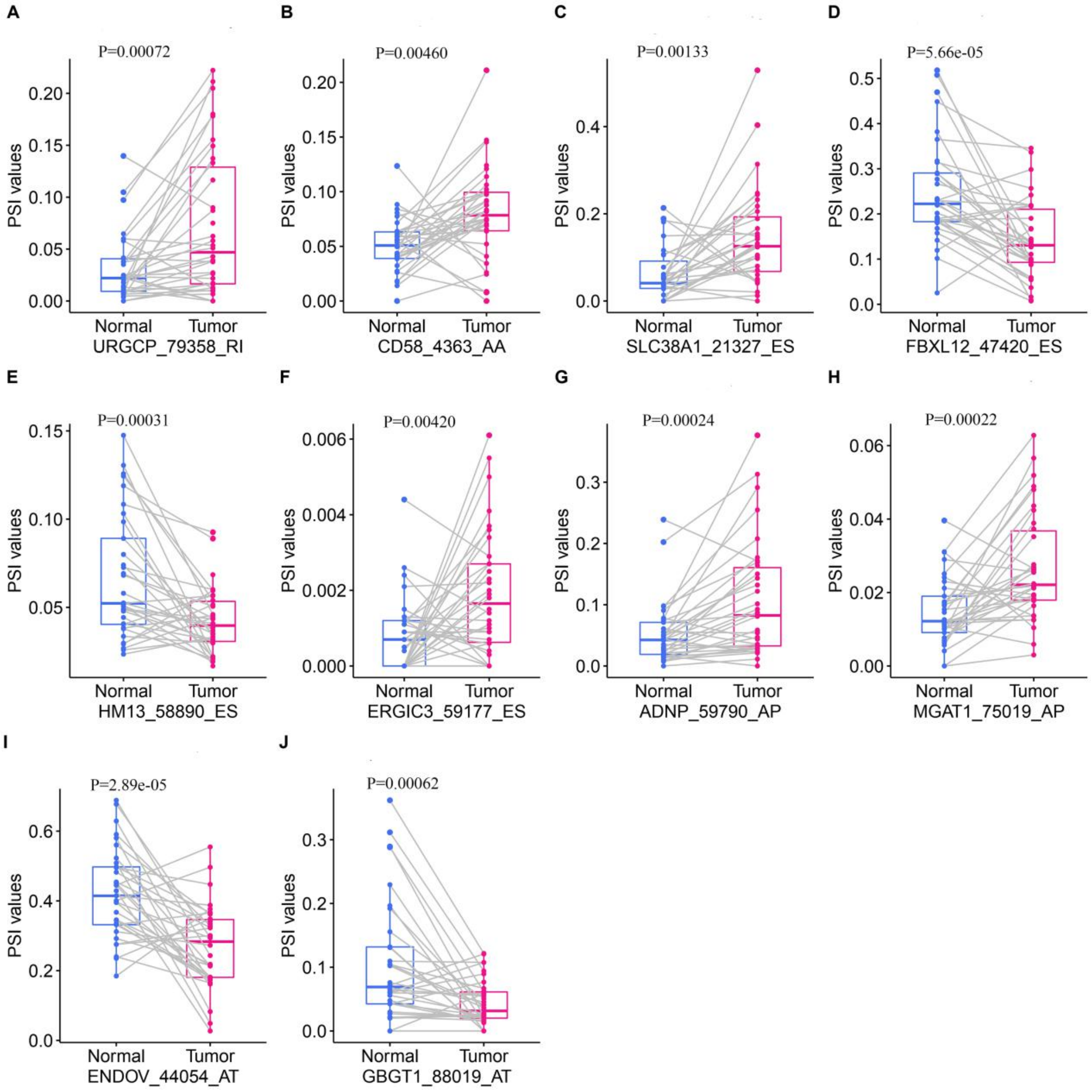

Figure S1: Expression of DEAS in 10 independent prognoses of gastric cancer.

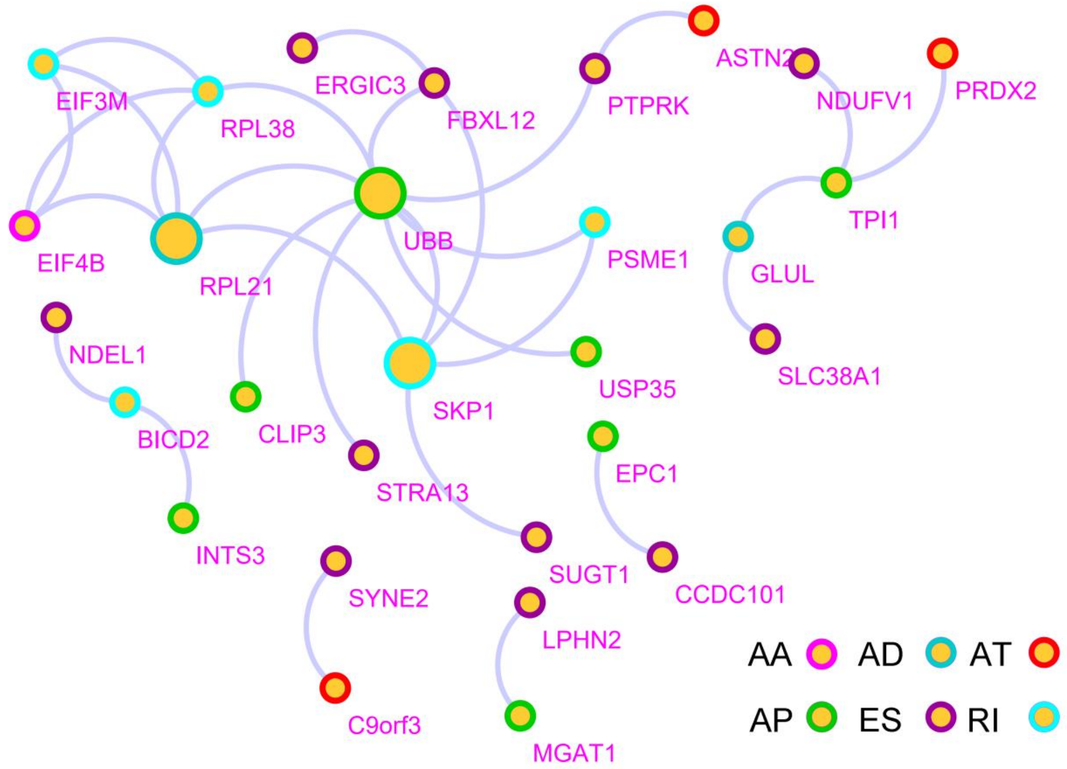

Figure S2: Protein-protein interaction network analysis of DEAS in gastric cancer. The color of the node represents the AS pattern. Hub nodes in the network are amplified.
